# Renal Cell Carcinoma Associated With HIV/AIDS: A Review of the Epidemiology, Risk Factors, Diagnosis, and Treatment

**DOI:** 10.3389/fonc.2022.872438

**Published:** 2022-04-01

**Authors:** Zhiqiang Zhu, Yihang Zhang, Hu Wang, Taiyi Jiang, Mengmeng Zhang, Yu Zhang, Bin Su, Ye Tian

**Affiliations:** ^1^ Department of Urology, Beijing Friendship Hospital, Capital Medical University, Beijing, China; ^2^ Department of Urology, Beijing Youan Hospital, Capital Medical University, Beijing, China; ^3^ Beijing Key Laboratory for HIV/AIDS Research, Clinical and Research Center for Infectious Diseases, Beijing Youan Hospital, Capital Medical University, Beijing, China

**Keywords:** renal cell carcinoma, HIV, AIDS, risk factors, diagnosis, treatment

## Abstract

Renal cell carcinoma (RCC), one of the most common genitourinary tumors, is induced by many factors, primarily smoking, obesity, and hypertension. As a non-acquired immunodeficiency syndrome (AIDS)-defining cancer, human immunodeficiency virus (HIV) may also play a critical role in the incidence and progression of RCC. It is evident that individuals who are infected with HIV are more likely than the general population to develop RCC. The age of RCC diagnosis among HIV-positive patients is younger than among HIV-negative individuals. However, many other characteristics remain unknown. With the increase in RCC incidence among HIV-infected patients, more research is being conducted to discover the relationship between RCC and HIV, especially with regard to HIV-induced immunodeficiency, diagnosis, and treatment. Unexpectedly, the majority of the literature suggests that there is no relationship between RCC and HIV-induced immunodeficiency. Nonetheless, differences in pathology, symptoms, or treatment in HIV-positive patients diagnosed with RCC are a focus. In this review, we summarize the association of RCC with HIV in terms of epidemiology, risk factors, diagnosis, and treatment.

## Introduction

Globally, renal cell carcinoma (RCC) is the 9^th^ most common cancer in men and the 14^th^ most common cancer in women (1). RCC derived from tubular epithelial cells is the most common cancer of the kidney, accounting for approximately 80% (2). The etiology of RCC remains unknown, and there are more than ten pathological classifications. In general, clear cell RCC (ccRCC) and nonclear cell RCC (nccRCC) are used to pathologically divide RCC into two main parts: ccRCC is the most common, accounting for 70%~75% of cases; nccRCC represents 15%~30% of cases (3). Risk factors for RCC are tobacco smoking, high body mass index (BMI, especially obesity), hypertension, occupational exposure, diet, and drug use (4). With the development of ultrasonography and imaging technology, many methods are used to detect renal tumors. Ultrasound (US), computed tomography (CT) and magnetic resonance imaging (MRI) are critical means of detection, and each has different clinical advantages and disadvantages. Surgery is the pillar of treatment for localized or locally advanced RCC and is the only curative treatment. Partial nephrectomy (PN) and radical nephrectomy (RN) are the main operations (5). Since 2005, multiple new drugs have been approved, including tyrosine kinase inhibitors (TKIs) and immune checkpoint inhibitors (ICIs). Hence, the overall survival (OS) of patients with metastatic RCC (mRCC) has increased from 1 year in the cytokine era to approximately 2.5~3 years in the TKI and immunotherapy eras (6). Despite major advances in exploring the molecular basis of RCC carcinogenesis, the selection of therapy is still based on clinical presentation and patient body status. Moreover, there are a number of concerns that should be considered for different populations, such as those with coinfection with human immunodeficiency virus (HIV).

HIV is responsible for human immunodeficiency syndrome (AIDS), which was discovered in 1983 (7). According to the UNAIDS, there were 1.5 million individuals in 2020 who were newly infected with HIV and 680,000 who died from AIDS-related illnesses. There are 37.7 million HIV-positive patients worldwide (8). Before the era of antiretroviral therapy (ART), people living with HIV/AIDS (PLWH) were at high risk of AIDS-related events, such as opportunistic infections and AIDS-defining malignancies, resulting in a short survival time. With the advent of ART, the lifespan of PLWH was prominently increased and even closed to that of HIV-negative populations, and the incidence of AIDS-defining cancers (ADCs) decreased dramatically (9–11). Nevertheless, non-AIDS-defining cancers (NADCs), including but not limited to lung, liver, kidney, anal and skin tumors, have gradually emerged (9, 12, 13). At present, RCC is regarded as an NADC and has partially different features in HIV-positive and HIV-negative populations. In this review, we focus on the epidemiology, risk factors, diagnosis and treatment of RCC patients with and without HIV.

## Morbidity and Mortality

During the last 2 decades, there has been an annual increase of 2% in the incidence of RCC worldwide and in Europe. Indeed, nearly 99,200 new RCC cases and 39,100 kidney cancer-related deaths were reported in the European Union in 2018 (5). As reported in 2022, the newest total estimated numbers of new kidney and pelvis cancer cases and deaths were 79,000 and 13,920, respectively, in the United States (14). There are no detailed data on how many RCC patients are coinfected with HIV or how many HIV-positive patients are diagnosed with RCC. Nevertheless, early literature reported that the HIV-positive population has an 8.5-fold greater chance of developing RCC than the general population and that the average age of occurrence is approximately 15 years younger (15). Other studies found the same conclusions, namely, that the HIV-positive population has a high incidence of RCC (16, 17). One meta-analysis of seven studies including 444,172 HIV/AIDS patients reported that the standardized incidence ratio of kidney cancer in people with HIV/AIDS was 1.50 (18). Overall, it is clear that HIV-positive patients are at a high risk of being diagnosed with RCC and that the age of these individuals is younger than that of the general population.

## Risk Factors

To date, the well-known risk factors for RCC are cigarette smoking, obesity, hypertension and the von Hippel-Lindau (VHL) mutation. Alcohol, occupational exposure, diet, drugs and caffeine have controversial effects in RCC (2, 19).

Smoking, especially cigarette smoking, is confirmed to promote the carcinogenesis of many tumors, including RCC. Tobacco smoke includes a mixture of carcinogens implicated in the etiology of RCC. In 2016, Cumberbatch et al. reported that the risk of developing RCC was significantly higher for all smokers than for nonsmokers. Among them, current smokers had a greater risk than former smokers (20). A meta-analysis of 24 studies reported a strong dose-dependent increase in the risk of RCC in both sexes. In addition, RCC patients who had quit smoking for >10 years had better outcomes than those who had quit smoking for 1-10 years (21). Some studies reported that smokers had worse outcomes in RCC than nonsmokers not only with respect to surgery but also targeted treatment (22–25). A recent cohort study indicated that heavy smoking (more than 40 pack-years) was associated with a significant increase in RCC incidence (26). Of course, smoking also has a negative effect on HIV/AIDS. The prevalence of smoking in individuals infected with HIV is higher than that in the general population. Additionally, these individuals are less likely to quit smoking than the general population (27, 28). Hence, smoking puts HIV-positive patients at risk of many of the known health consequences, and these patients evidently have increased morbidity and mortality related to smoking (29). Although the prevalence of RCC patients with HIV is unknown, we find that HIV-positive patients who are current smokers or ever smokers have a greater tendency to be diagnosed with RCC. As smoking cessation is less likely, HIV-positive patients might have a worse outcome than HIV-negative patients diagnosed with RCC. Therefore, encouraging PLWH to stop smoking will affect the prognosis of these individuals, especially with cessation of smoking for more than 10 years.

Obesity is another evident risk factor for RCC at present (30). BMI, defined as weight (kg) divided by the square of body height (m), is often used to assess body mass. Obesity is defined as BMI of 30 kg/m^2^ or greater in non-Asian populations and 25 kg/m^2^ or greater in Asian populations. A meta-analysis including 17 epidemiological studies estimated a 24% increase in the risk of developing RCC in men and a 34% increase in women for each 5-point increase in BMI (31). Another meta-analysis showed a significant association between excess body weight and increased risk of RCC in both men and women, with a slightly higher risk in women. In addition, the researchers found that each 1-kg/m^2^ increase in BMI corresponded to a 4% increase in the risk of RCC (32). However, among studies about RCC and obesity, there were some viewpoints regarding obesity as a protective factor called the “obesity paradox” (33). One clinical-based cohort and meta-analysis of 1,543 patients who underwent nephrectomy for RCC in Korea indicated that high BMI prior to renal surgery was associated with improved OS, cancer-specific survival (CSS) and recurrence-free survival (RFS) when compared with low BMI (34). Recent studies similarly reported that high BMI might play a positive role in RCC compared with normal or low BMI (35–37). Turco et al. reviewed this phenomenon and offered an explanation, suggesting that these studies considering BMI as a protective factor in RCC had some limitations. BMI is used to assess body weight conveniently, but it does not accurately reflect the respective weight of fat, muscle, and bone mass. Similarly, it also does not assess fat in the subcutaneous area or viscera. Some studies define obesity as BMI of more than 25 kg/m^2^ instead of 30 kg/m^2^, amplifying the inclusion criteria. Other possible reasons, such as nutrition and genetic and molecular features, might be associated with the obesity paradox (33). For HIV-positive patients, obesity is also a factor to focus on due to ART use, and unhealthy diet and low exercise might affect body weight. A recent report indicated that obesity and overweight were common in older patients with HIV and associated with the presence of metabolic disease and multimorbidity (38). Overall, obesity is regarded as a risk factor in multiple diseases. HIV patients should maintain strict control of their body weight to not only prevent the development of RCC but also to reduce the risk of other metabolic diseases.

Hypertension is a significant risk factor for both kidney cancer incidence and mortality in men, as revealed by multivariable regression analysis (39). In the VITAL study involving a prospective cohort of more than 77,000 US residents, hypertension was independently associated with the risk of RCC (40). In addition, there was evidence indicating that hypertension might have a dose-dependent effect on kidney cancer risk. A recent meta-analysis of 18 prospective studies showed that each 10-mmHg increase in blood pressure was associated with a 10%~22% increase in the risk of kidney cancer (41). The prevalence of hypertension in HIV-infected patients is higher (42), and there are many factors that induce hypertension. A recent meta-analysis showed that exposure to ART was associated with a significantly increased risk of hypertension (43). Given that ART is used throughout the life of HIV-infected patients, blood pressure should be examined regularly, especially in older patients. Controlling blood pressure within a certain range is an effective way to reduce morbidity and mortality in HIV-infected patients with RCC. However, the optimal blood pressure range and which antihypertensive drugs should be chosen need further investigation.

For HIV-positive patients, CD4^+^ T cell count is an extremely important factor. ADCs are strongly associated with immunosuppression (17), especially when CD4^+^ T cell counts decrease by 200 cells/μL. However, not all NADCs are associated with immunosuppression (44), and HIV-induced immunosuppression appears to play a lesser role than lifestyle habits and viral coinfection compared with those in ADCs (45). Some related reports are described below. In 1990, Adjiman et al. reported a 25-year-old patient diagnosed with RCC associated with advanced malignant lymphoma, which is known to be directly related to immune depression (46). Azon-Masoliver et al. also reported a patient with both Kaposi’s sarcoma and renal cell adenocarcinoma. These two cases seem to indicate that RCC may have an association with immunodeficiency. However, given that only two patients were described, it is not possible to determine whether a relationship between immunodeficiency and RCC exists. In 1997, Stephen A. Baynham et al. reported that RCC may occur in individuals with higher CD4 T cell counts and that the occurrence of RCC might not be only due to nonspecific immunosuppression, as seen with AIDS-related lymphoma (15). In 2008, Bruce J. Dezube et al. reported nine HIV-positive patients who were diagnosed with RCC, 7 of whom had mild-to-moderate immunosuppression (CD4 T cell count: 62~731 cells/μL). The authors concluded that HIV-related immunosuppression might not play an important role in RCC. Instead, HIV infection and ART might result in nephropathy and diabetes, both of which are potential risk factors for RCC (45). In the same year, Annah B. Layman et al. reported no association between CD4 T cell count at AIDS onset and the risk of RCC during the incidence period (47). In 2016, Wee Loon ONG et al. reported seven HIV-positive patients diagnosed with RCC; most had a mild-to-moderate immunodeficiency (CD4^+^ T cell counts: 178~1,352 cells/μL). Additionally, five of the patients had viral loads below 50 copies/mL (48). These reports appear to suggest no association between RCC and HIV-induced immunosuppression. A similar conclusion was reached in 2021. Zhang and Zhu et al. from Beijing Youan Hospital reported 19 HIV-infected patients diagnosed with RCC. They concluded that there was no evidence to support a relationship between immune deficiency and tumor progression, even though some patients did not undergo regular ART (49). Overall, recent studies have tended to consider that there is insufficient evidence to prove an association between HIV-induced immunodeficiency and RCC. However, there were many limitations in these studies. First, the number of RCC patients with HIV infection was relatively low. Second, most of the patients were men. Third, we suspect that RCC has an association with HIV-induced immunodeficiency, but patients with low CD4 T cell counts tend to be diagnosed with ADC and have a worse prognosis; thus, they may die because of ADCs and opportunistic infections at a younger age before they develop RCC. Fourth, there was a lack of different ranges of CD4 T cell counts to evaluate the association with immunodeficiency and RCC. Therefore, further studies are needed.

The VHL gene, located at chromosome 3p, is a tumor-suppressor gene that plays an important role in the development of RCC (50). VHL is not only the most frequently studied gene but also has the highest mutation prevalence, accounting for 64% (51). Mutant VHL lacks the ability to target hypoxia-inducible factor (HIF) involved in angiogenesis and mitogenesis for destruction by the pVHL-E3 ligase complex ubiquitin-proteasome pathway (52). Interestingly, stabilization and increased transcription and expression of HIF-1 are clearly affected by human oncogenic viruses by disrupting degradation of HIF-1 (53). Moreover, one study indicated that proper pVHL increased HIV-1 replication and gene expression. Researchers have also found that the Cul2/VHL-mediated degradation pathway promoted integrase (a key enzyme in the HIV integration process) stabilization in RCC4 cells (54). Hence, HIV might actually participate in the development of RCC. Besides, we speculate that in RCC patients with VHL gene mutation, HIV replication can, to some degree, be influenced by a reduction in pVHL expression *in vivo*. That may become a new target of treatment to diminish HIV after we know for sure how pVHL functions in HIV replication. In addition, other modifiable risk factors, including alcohol consumption, caffeine, diet, occupational exposure and drugs, are more or less associated with RCC. Further investigations are needed.

## Diagnosis

### Clinical Presentation

The major clinical presentations, or classical triad, described in RCC are hematuria, flank pain and abdominal or flank mass, but they are only seen in a few individuals. Other clinical presentations, such as weight loss, acute varicocele and symptoms due to metastasis, are found in some patients (55). HIV-positive populations with RCC, in addition to having parallel symptoms, may exhibit some AIDS-related clinical manifestations, such as opportunistic infections and ADCs, especially in those who have low CD4^+^ T cell counts.

### Imaging

US, CT, and MRI are used to screen for RCC (56). US is one of the first methods used for the diagnostic imaging of renal lesions, as it is easily repeatable, does not require radiation and is cost-effective. Hence, US is a readily available, fast and easy method of evaluation for clinicians. However, it requires operator experience, and the kidneys cannot always be satisfactorily imaged (57). Moreover, the use of US to screen for renal cancer in asymptomatic patients is controversial, as the rate of incidental malignant findings has been found to be very low, at only 0.2% (58). One study found that CT was a better choice than US when the diameters of renal lesions were 0 to 5 mm (the detection rates were 47% and 0%, respectively), and the detection rate increased with an increase in lesion diameter. For instance, in large lesions (10 to 35 mm), the detection rates were 80% for CT and 82% for US (59). Despite its limited sensitivity for small lesions, US may be useful to determine whether a lesion is likely to be cystic in nature but appears hyperdense on a CT scan in patients in whom contrast agents are contraindicated. Regardless, US is still an important method for the detection and diagnosis of RCC. CT has been the gold standard for cross-sectional RCC imaging since the 1990s. Due to the increased vascularity in RCC, it might be better visualized with contrast-enhanced CT (57). Nevertheless, CT has some limitations that restrict its wide use. Contrast-enhanced CT is not recommended for patients who are allergic to contrast agents, those who are pregnant, and those who are undergoing renal dialysis. MRI has played an increasingly important role in imaging patients with RCC, particularly those who are intolerant to CT (57). According to the American College of Radiology, MRI is comparable to CT for RCC staging and post-treatment follow-up and for the evaluation of indeterminate renal masses (60). There is an evidence that MRI may better evaluate renal masses previously deemed indeterminate on CT imaging or US (61). In addition, a report showed that MRI imaging has a sensitivity of 92.3% and a specificity of 86.4% in the diagnosis of inferior vena cava thrombus before surgery (62). Regardless, MRI has some limitations, such as high cost, inconvenience, and a long examination time. Each imaging technique has different advantages and shortcomings for the diagnosis of RCC. If patients have symptoms or renal masses found through certain examinations, excluding any contraindications, CT as the gold standard is strongly recommended. CT is more sensitive than US, especially in the detection of small renal masses. If patients have contraindications for CT, MRI is another method that can be used. Considering the advantages and disadvantages of each imaging technique, multiple imaging methods have been combined in the field of RCC diagnosis, thus improving the sensitivity and accuracy.

### Histological Diagnosis

According to the WHO classification of tumors of the kidney in 2016, renal cell carcinoma is divided into 16 subtypes. The most common subtype is ccRCC, accounting for 70%~75% of cases. Papillary RCC (pRCC) is the most common non-ccRCC subtype, accounting for 15%~20% of cases. Chromophobe RCC (chRCC) and other pathological types account for 5% each (3). There are considerable differences in tumor stage, grade, and CSS between each type. Each pathological type has different molecular features and immunohistochemistry profiles. For example, ccRCC is characterized by cells with clear cytoplasm and a delicate capillary network. However, infiltrative growth, eosinophilic cytoplasm or globules, poorly differentiated adenocarcinoma-like morphology, rare papillary formation, giant multinucleated tumor cells, and sarcomatoid/rhabdoid morphology are characteristics of ccRCC subtypes. Some of them are related to a worse prognosis and may serve as biomarkers of prognosis (63). With the use of more imaging techniques, early-stage RCC can be detected incidentally, improving the cure rate and survival time of patients. Some case reports showed that there were no marked differences in pathology between RCC with and without HIV (45, 48, 49). In 2008, Gaughan et al. reported nine RCC patients with HIV, six of whom had ccRCC (45). In 2016, Wee Loon ONG et al. reported 7 RCC patients diagnosed with HIV, and five had ccRCC (48). In 2021, Zhang and Zhu et al. reported nineteen patients diagnosed with RCC and HIV; seventeen of these patients were diagnosed with ccRCC, accounting for 89%. One patient had partial ccRCC and partial pRCC, and another had chRCC (49). According to these case reports, ccRCC is still a dominant pathological type in HIV-infected patients.

## Treatment

Currently, localized RCC can be treated by PN or RN (5). As a refractory tumor, the optimal treatment of mRCC has been constantly explored. Given the poor response of RCC to radiation and chemotherapy, targeted treatment and immunotherapy are commonly used for mRCC, with good results for the majority of patients (2, 64, 65). In addition, the combination of cabozantinib and nivolumab is now recommended as the first-line treatment of advanced disease, bringing new hope to RCC patients (66).

Zhang and Zhu et al. reported that the treatment approaches appear to be the same for HIV-positive and HIV-uninfected RCC patients, and their prognosis following PN is no worse than that of patients undergoing RN. In a retrospective study of 19 patients, 12 with varying degrees of immunodeficiency (CD4^+^ T cell counts < 400 cells/μL) were alive at the 34-month posttreatment follow-up, with only one case of metastasis. In general, additional trials are still needed to evaluate the effect of immunodeficiency on RCC recurrence and metastasis in HIV-positive individuals (49). Similar to the aforementioned study, an article from Australia suggested that patients with RCC and HIV infection should be given the same treatment measures as the general population (48). However, neither study found an association between immunodeficiency and tumor progression in HIV-infected patients.

RCC is an immune-responsive tumor, and with the emergence of ICIs, there is new hope for the treatment of advanced RCC (67–69). Recently, the phase III KEYNOTE-426 study showed that pembrolizumab (targeting PD-1) plus axitinib continues to result in superior clinical outcomes versus sunitinib, and these results provide further evidence that using pembrolizumab plus axitinib as first-line therapy as the standard of care for advanced RCC is an option that benefits patients (70). In fact, the immune checkpoint PD-1 not only serves as a therapeutic target for RCC but also plays a role in the body’s fight against HIV. More interestingly, studies have demonstrated that PD-1 expression and exhaustion occur in HIV-specific CD4^+^ and CD8^+^ T cells and that PD-1 expression is associated with viral load, CD4 T cell count, and cytotoxic function of CD8^+^ T cells (71–74). This PD-1 expression and T cell depletion can be reduced by ART but not to pre-HIV infection levels (75). Therefore, ART should not be interrupted during RCC treatment. More recently, Li et al. noted that CD8^+^ T cell activity can be restored by targeting the adenosine and PD-1 signaling pathways together. Further study revealed that targeting both the CD39/adenosine and PD-1 pathways improved CD8^+^ T cell antiviral effectiveness more than targeting only one immune checkpoint pathway, which can be a potential strategy for treating HIV (76). Similarly, other immune checkpoints may play an important role during HIV infection, such as CTLA-4, TIM-3, TIGIT, and LAG-3, which are all associated with changes in some patient indicators during HIV infection (74, 77–80). In a recent study, investigators evaluated the effect of intravenous pembrolizumab every 3 weeks on HIV latency in 32 PLWH and patients with cancer. The findings support the use of anti-PD-1 therapy in combination with other therapeutic approaches to reduce the HIV viral reservoir, with fresh perspectives on ICI use for HIV infection (81).

For advanced cancer patients with HIV, both the feasibility and safety of ICIs have been demonstrated in two clinical investigations. In the phase I study Cancer Immunotherapies Network Study-12 (CITN-12), the investigators recruited HIV-infected patients with advanced cancer who had CD4 T cell counts greater than or equal to 100 cells/μL, underwent ART for 4 weeks or more, and had an HIV viral load less than 200 copies/mL. Interestingly, the clinical benefit rate (defined as tumor shrinkage or stabilization at 24 weeks) for pembrolizumab was 17%, and the toxicity profile of the ICIs was similar to that of HIV-uninfected individuals (82). In addition, the phase 2 DURVAST study, which aimed to assess the feasibility and safety of durvalumab for the treatment of solid tumors in PLWH, observed partial responses in 4 of 16 evaluable patients (25%). Five patients (31%) had stable disease, and 4 of them had durable stable disease (50% disease control) without unexpected toxicity (83).

PD-1 inhibitors have great potential in the management of mRCC and at the same time modulate potential immunosuppression in PLWH. However, most clinical trials on ICI treatment for cancer have not included PLWH (84), which prevents them from acquiring the same cancer treatment opportunities as those who are not infected with HIV, despite their higher risk of developing cancer and their higher cancer-specific mortality (85, 86). Initially, PLWH were excluded from clinical trials, possibly due to the lack of consistent evidence-based guidelines for the development of relevant clinical trials and concerns about some potential risks arising from interactions between ICIs or other drugs and ART drugs (87). However, with growing evidence that ICIs have similar efficacy and tolerability in PLWH compared to the general population in advanced cancer treatment (88, 89), in 2020, the Food and Drug Administration (FDA) recommended that PLWH with acceptable immune function be included in cancer trials. In the future, more clinical trials, such as NCT04514484, which includes PLWH with advanced RCC, should be conducted to bring more survival possibilities to this group of patients.

Of course, some kinds of conventional drugs for HIV/AIDS may have effects on ccRCC when combined with anti-ccRCC drugs. For example, non-nucleoside reverse transcriptase inhibitors such as efavirenz and nevirapine can either induce reversible downregulation of cell proliferation or enhance cell differentiation in human renal carcinoma cells (90). Moreover, the protease inhibitors lopinavir and nelfinavir used for HIV/AIDS treatment substantially improve the activity of carfilzomib in ccRCC cell lines and primary cells at therapeutically relevant drug concentrations (91). These studies provide a different view that traditional drugs for both HIV/AIDS and RCC may have synergistic effects and even become proper regimens.

## Conclusions

RCC is one of the most common kidney cancers. People with the risk factors smoking, obesity, and hypertension are at high risk of RCC. The symptoms of RCC are not obvious in the majority of patients, especially in the early stage of the tumor. Hence, regular examinations are needed in high-risk groups. Three methods are most commonly used clinically for detection: US, CT and MRI. Each of them has advantages and shortcomings. Their combination can improve the sensitivity and accuracy of the diagnosis of RCC. To date, surgery is still the only way to cure RCC. However, with the development of understanding of etiology, targeted treatments and immunotherapies have continued to emerge. Moreover, there is an increasing number of treatments for RCC, especially mRCC. PLWH are at high risk of RCC as well, with a younger age of onset. We still do not understand why these special groups tend to develop RCC. According to some case reports, there are no differences in pathological type, clinical presentation, screening method, and treatment compared with the general population. Interestingly, most reports indicate that no association between RCC and HIV-induced immunodeficiency. Therefore, imaging examinations in HIV-infected patients are critically needed, even in those with high CD4^+^ T cell counts. Surgical treatment is strongly recommended for patients with localized RCC with HIV/AIDS. RN tends to be offered to patients with lower CD4^+^ T cell counts. Although the combination of targeted treatment and immunotherapy has emerged, bringing new hope for mRCC patients, there is no clear evidence of the optimal treatment for mRCC patients with HIV/AIDS. Consequently, advanced investigations are urgently needed, and more treatments need to be developed.

## Author Contributions

BS and YT conceived and supervised the whole study, TYJ, MMZ, and YZ. searched the literature, selected studies and provided important scientific input, ZQZ, YHZ, HW, and BS wrote the draft of the manuscript. All authors listed, have made a substantial, direct, and intellectual contribution to the work. All authors read and approved the final manuscript.

## Funding

This work was supported by the National Natural Science Foundation of China (NSFC, 81974303 and 81772165), the “Climbing the peak (Dengfeng)” Talent Training Program of Beijing Hospitals Authority (DFL20191701), the National 13^th^ Five-Year Grand Program on Key Infectious Disease Control (2017ZX10202102-005-003 and 2017ZX10202101-004-001), and the Beijing Key Laboratory for HIV/AIDS Research (BZ0089). The funders had no role in study design, data collection and analysis, decision to publish, or preparation of the manuscript.

## Conflict of Interest

The authors declare that the research was conducted in the absence of any commercial or financial relationships that could be construed as a potential conflict of interest.

## Publisher’s Note

All claims expressed in this article are solely those of the authors and do not necessarily represent those of their affiliated organizations, or those of the publisher, the editors and the reviewers. Any product that may be evaluated in this article, or claim that may be made by its manufacturer, is not guaranteed or endorsed by the publisher.
